# Seasonal variation in the abundance and distribution of *Anomalocardia flexuosa* (Mollusca, Bivalvia, Veneridae) in an estuarine intertidal plain

**DOI:** 10.7717/peerj.4332

**Published:** 2018-02-27

**Authors:** Jacqueline S. Silva-Cavalcanti, Monica F. Costa, Luis H.B. Alves

**Affiliations:** 1 Unidade Academica do Cabo de Santo Agostinho (UACSA), Universidade Federal Rural de Pernambuco, Cabo de Santo Agostinho, Pernambuco, Brazil, Pernambuco, Brazil; 2 Departamento de Oceanografia, Universidade Federal de Pernambuco, Recife, Pernambuco, Brasil, Pernambuco, Brazil

**Keywords:** Traditional fisheries, Population dynamics, Size classes, *Anomalocardia brasiliana*, Estuarine ecology

## Abstract

Spatial and temporal density and biomass of the infaunal mollusk *Anomalocardia flexuosa* (Linnaeus, 1767) evaluated a tidal plain at Goiana estuary (Northeast Brazil). Three hundred and sixty core samples were taken during an annual cycle from three intertidal habitats (A, B and C). Shell ranged from 2.20 to 28.48 mm (15.08 ± 4.08 mm). Recruitment occurred more intensely from January to March. Total (0–1,129 g m^−2^) differed seasons (rainy and dry), with highest values in the early rainy season (221.0 ± 231.44 g m^−2^); and lowest values in the late dry season (57.34 ± 97 g m^−2^). The lowest occurred during the late rainy (319 ± 259 ind m^−2^) and early dry (496 ± 607 ind m^−2^) seasons. Extreme environmental situations (e.g., river flow, salinity and water temperature) at the end of each season also affected density ranges (late dry: 0–5,798 ind m^−2^; late rainy: 0–1,170 ind m^−2^). *A. flexuosa* in the Goiana estuary presented a dominance of juvenile individuals (shell length < 20 mm), with high biomass main the recruitment period. Average shell length, density and biomass values suggest overfishing of the stock unit. *A. flexuosa* is an important food and income resource along its whole distribution range. The species was previously also known as *Anomalocardia brasiliana* (Gmelin, 1791).

## Introduction

Estuarine intertidal areas support a large number of clam species of varying ecologic guilds, and therefore have been used by humans as important fishing grounds for millenia. Edible bivalves are widely collected around the World and became the basis of many coastal communities’ livelihoods (Silva-Cavalcanti & Costa, 2011). On the tropical and sub-tropical littoral of the Eastern South America and Caribbean, *Anomalocardia flexuosa* (Linnaeus, 1767), an infaunal clam distributed from the Western Indies to Uruguay (Rios, 1985), is one of the main shellfish fisheries product, especially from the 19th century. *A. flexuosa* is an important food and income resource along its whole distribution range. The species was previously also known as *Anomalocardia brasiliana* (Gmelin, 1791), a junior synonym. *Anomalocardia brasiliana* was recognized as a synonym of a *A. flexuosa* by Fischer-Piette & Vukadinovic (1977: 45ff). The synonymy was followed by subsequent authors such as Huber (2010: 719).

Biology, ecology and ethnobiology studies about *Anomalocardia flexuosa* started in 1970s. Important ecological studies were published for São Paulo (Narchi, 1976; Schaeffer-Novelli, 1976; Arruda-Soares, Schaeffer-Novelli & Mandelli, 1982; Leonel, Magalhães & Lunneta, 1983; Corte et al., 2015), Santa Catarina (Pezzuto & Echternacht, 1999; Boehs & Magalhães, 2004; Boehs, Absher & Cruz-Kaled, 2008), Paraíba (Grotta & Lunetta, 1980), Ceará (Araújo & Rocha-Barreira, 2004; Barreira & Araújo, 2005), Pernambuco (Oliveira et al., 2013) and Rio Grande do Norte (Rodrigues et al., 2008), covering a climate regimes. Living populations of *A. flexuosa* from the Caribbean were also (Monti, Frenkiel & Möueza, 1991; Mouëza, Gros & Frenkiel, 1999). All these studies described the reproductive biology of *A. flexuosa*, and explored the biotic and abiotic variables that control the spatio-temporal of size, density and biomass.

Species living in intertidal habitats present spatio-temporal resulting from interactions between physical (tidal amplitude, sub-aereal exposure, water salinity and substratum) and biological (predation, competition, fisheries and other) factors (Rodil et al., 2008; Magalhães, Freitas & Montaudouin, 2016; Gerwing et al., 2016). Moreover, other variables can influence the distribution of these organisms, as reproductive behaviour, food availability, hydrodynamics, organic matter in the sediments and a number of combinations among them (Araújo & Rocha-Barreira, 2004; Gerwing et al., 2016). In addition, human interventions (fisheries) are also a factor that deeply influences the population dynamics of edible benthic bivalves. In some cases, a population might be exploited for long periods (centuries) and, occasionally, become over-exploited, which will compromise its resilience. This is probably the case of *A. flexuosa* at many sites (Rodrigues et al., 2008).

Knowledge of spatio-temporal distribution and ecology of these bivalves are basis for the establishment of managed fisheries (including traditional) and environmental management actions; therefore, can favour the maintenance of natural stocks and contribute for the sustainable exploitation of this resource (Araújo, 2001). Often, studies are published based on the dynamics of higly-disturbed population, without comparisons with control-sites. This might make the interpretation and of ecological data more difficult for use in management decisions.

The present study aimed to evaluating the spatio-temporal distribution of *Anomalocardia flexuosa* at Goiana estuary (Northeast Brazil) during an annual cycle. We considering shell length, density and total biomass in this evaluation. Our hypothesis is that fluctuation of environmental parameters (e.g., salinity, grain size) affects a heavily exploited population of *Anomalocardia flexuosa* distribution and other biological variables at different spatial and time (seasons).

## Material and Methods

### Study site and sampling

**Figure 1:**
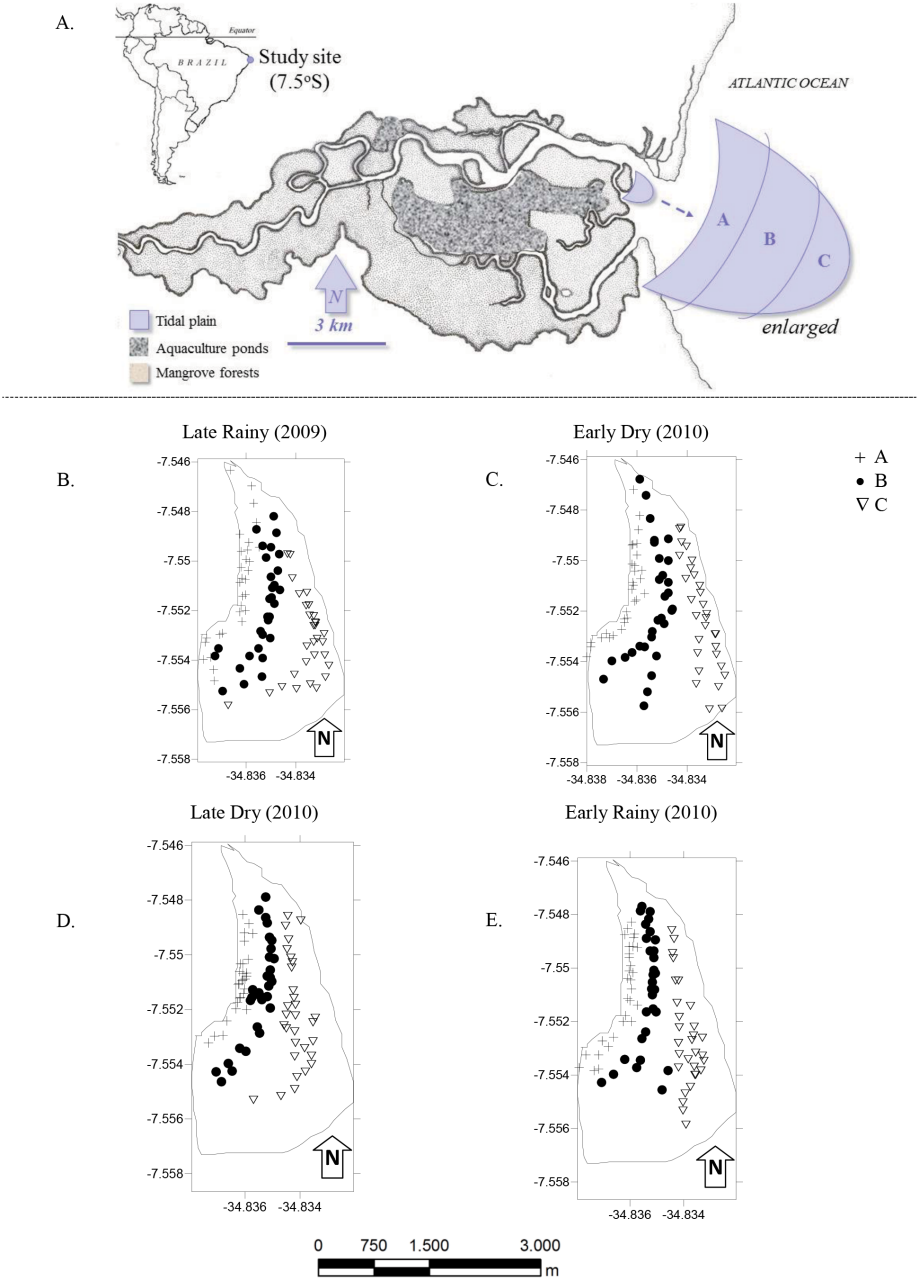
Study area. (A) Location of the Goiana estuary and position of the tidal plain within the estuary. (B–E) Distribution of cores (*N* = 360) according to season ((B) Late Rainy; (C) Early Dry; (D) Late Dry and (E) Early Rainy) and habitats (habitats A, B and C).

The Goiana estuary (7°S, 35°W) has a length of about 25 km of main channel with an average river discharge of ∼12 m^3^ s^−1^. At the lower estuary (Fig. 1A), water (24–27 °C) and air (∼25 °C) show little variation. Salinity ranges between 8 (upper estuary) and 36 (lower estuary) (Dantas et al., 2010) (Fig. 1), depending on rainfall and river flow. In 2007, the estuary became part of a marine protected area (MPA) that covers 4,700 ha of the Extractive Reserve (RESEX-Acaú/Goiana) that including the river’s main channel, flooded mangrove forests, coastal habitats (beaches; tidal plains) and fishers’ settlements (Barletta & Costa, 2009).

The most intense and frequently harvested tidal plain (Silva-Cavalcanti & Costa, 2009) was chosen for this study (Fig. 1A). It lies right in the center of the RESEX, with easy access to all its participating communities. Tidal plain has an exposed area of ∼3.7 km^2^ at the lowest tides (−0.1), and was divided in three sampling habitats: A, nearer to the mangrove forest and mostly exposed during low tide; B was on the top of the tidal plain and always exposed during low tide and; C at the fringe of the tidal plain and only occasionally exposed at low tide (Table 1).

The monthly sampling took place during spring tides (new moon), and followed a seasonal criteria previously established of early rainy season (March to May), late rainy season (June to August), early dry season (September to November) and late dry season (December to February) (Barletta & Costa, 2009).

Populational parameters variations were measured from samples taken with a cylindrical corer (∅ 15 cm, 20 cm height). Cores were distributed within each habitat (*N* = 10), and herefore 360 holes were obtained for this study (Fig. 1B). The area of the corer was *A* = (3.14) × *h*^2^. The total area sampled was 176 cm^−2^ or 0.0176 m^−2^. This value was used for Biomass and Density calculations.

### Abiotic data

For each sample, interstitial water was extracted, filtered and salinity was measured with a refractometer. Sub-samples of the sediments were oven-dried at 60 °C, and analysed for grain size (Suguio, 1973; Camargo, 2006). Fine sediments represented less than 5% of the material. Thus, there was not further analysis of this fraction. Analysis of variance (Two-way ANOVA) was used to test the effect of the factors time (season) and space (habitat) on interstitial salinity and grain size distribution. An *a posteriori* Bonferroni test was used to evidence which samples were significantly different in time (season) and space (habitat).

### Biotic data

Core samples were washed (1 mm mesh size); *Anomalocardia flexuosa* individuals separated and shell length (anteroposterior axis) of each specimen was measured using a digital caliper rule (±0.01 mm). To estimate Biomass, individuals were weighed (±0.001 g) whole to obtain wet weight and, after 24 h at 60 °C, weighing was reapeted for dry weight (total biomass and biomass ≥ 20 mm). Total Biomass (TB) was calculated using the mean dry weight of the total sampled population. For the purposes of this study, adult individuals were considered those with shell length ≥20 mm (Barreira & Araújo, 2005). Meat dry weight for individuals with shell length > 10 mm was used to calculate the condition Index (CI) and Meat Yield (MY) (Boehs, Absher & Cruz-Kaled, 2008). Field experiments were approved by the Federal University of Pernambuco and Enviroment Ministry of Brazil (MMA-IBAMA), IBAMA/Licença no 21096-1 (2008–2011).

The ANOVA tested for significant differences of shell length and biomass along space (habits A, B and C) and time (seasons). Where ANOVA showed a significant difference, an *a posteriori* Tukey’s HDS test was used to determine which means were significantly different at the 0.05 level of probability. The Kruskal-Wallis non-parametric analysis of variance was used for density analysis. All analyses used STATISTICA (version 8, 2008; StatSoft, Tulsa, OK, USA).

## Results

### Abiotic data

**Figure 2:**
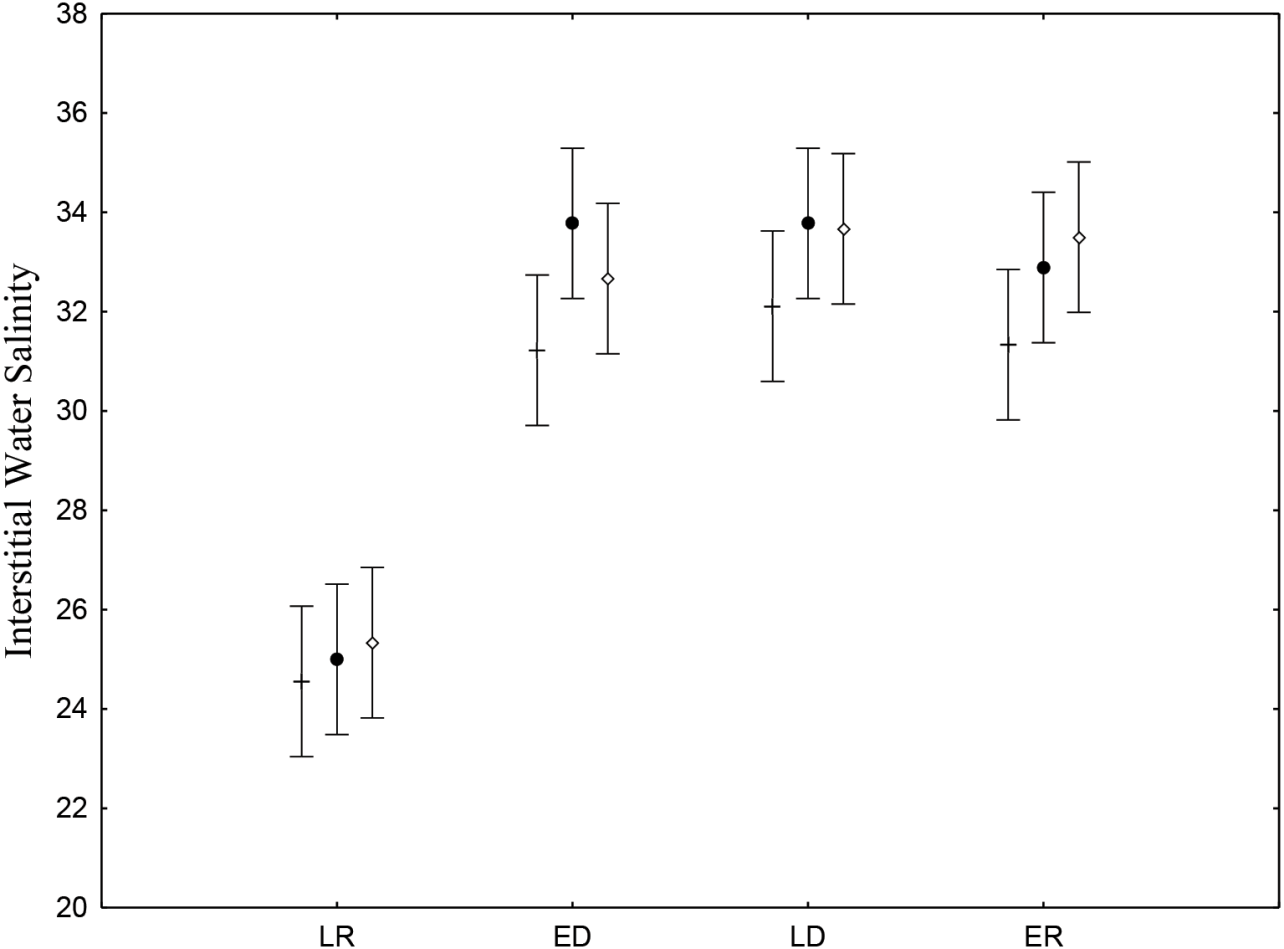
Salinity of sediments interstitial water. Average and standard deviations salinity of sediments interstitial water on the tidal plain of the Goiana estuary during different seasons (LR, Late Rainy; ED, Early Dry; LD, Late Dry, ER, Early Rainy) and habitats (+ = *A*; • = *B*; ◊ = *C*). Interaction between season *vs.* habitat: *F*_(6,96)_ = 0.47035, *p* = 0.82878.

Interstitial salinity was lower during the late rainy season (24.9 ± 0.71), when there is intense rainfall and greater volumes of riverwater reach the low estuary (Table 2). The late rainy season (June –August) presented significant differences (*p* < 0.05) (Fig. 2). Average water salinity was 32.5, 33.2 and 32.7 for the early dry, late dry and early rainy seasons, respectively. Interstitial water salinity did not present significant differences (*p* ≥ 0.05) when the three different are considered.

**Table 1:** Characteristics of each habitat sampled on the tidal plain of the Goiana estuary most used by mussel fishers.

Habitat	Main characteristics
A	Nearer to the mangrove forest and mostly exposed at low tide; average altitude 12 m (7 to 16 m) and 750 m wide; *Crassostrea rhizhopharae* shells buried in sand; Little *Anomaocardia flexuosa* fisheries.
B	Intermediate area and always exposed during low tide; Average 13 m (10 to 16 m) and 900 m wide; Presence of *Halodule wrightii* (seagrass); Intense *Anomalocardia flexuosa* fisheries.
C	Fringe of the tidal plain and only occasionally exposed during low tide; average altitude 11 m (3 to 16 m) and 600 m wide; *Crassostrea rhizhopharae* shells buried in sand; Some *Anomalocardia flexuosa* fisheries;

**Table 2:** Salinity of interstitial water of tidal plain sediments at Goiana estuary at the different habitats (A, B and C) and seasons (*n* = 9).

Season	Average ± stdev.		
	A	B	C
Late rainy	24.5 ± 3.13 (*n* = 9)	25.0 ± 3.39 (*n* = 9)	25.3 ± 2.12 (*n* = 9)
Early dry	31.2 ± 2.68 (*n* = 9)	33.7 ± 1.99 (*n* = 9)	32.6 ± 2.00 (*n* = 9)
Late dry	32.1 ± 1.45 (*n* = 9)	33.7 ± 1.48 (*n* = 9)	33.6 ± 2.06 (*n* = 9)
Early rainy	31.3 ± 2.30 (*n* = 9)	32.8 ± 2.37 (*n* = 9)	33.5 ± 1.58 (*n* = 9)

Significant differences in relation to spatial grain size distribution were not observed. However, there were significant spatial differences among seasons. The late rainy season was different (*p* < 0.05) from the others. The interaction habitat *vs*. season was not significant (*p* ≥ 0.05). All habitats (A, B and C) presented dominance of sand, except int the early rainy season (Table 3).

**Table 3:** Tidal plain Goiana estuary average sediment characteristics during the different seasons.

Season	Area	Grain size (%)			Sorting	Size scale	Φ
		Gravel	Sand	Silt / clay			
Late Rainy	A	1.57	93.68	4.74	Poorly sorted	Fine sand	2.35
		0.31	95.4	4.29	Moderately sorted	Fine sand	2.90
		0.67	92.95	6.38	Moderately sorted	Fine sand	3.02
		2.26	96.73	1.00	Moderately sorted	Medium sand	1.70
		6.00	90.12	3.87	Poorly sorted	Medium sand	1.75
		2.06	96.18	1.76	Moderately sorted	Medium sand	1.91
		1.42	92.56	6.02	Moderately sorted	Fine sand	2.74
		3.09	95.68	1.23	Poorly sorted	Medium sand	1.23
		1.60	95.97	2.43	Moderately sorted	Medium sand	1.89
	B	2.69	92.98	4.32	Poorly sorted	Medium sand	1.95
		1.49	94.84	3.66	Poorly sorted	Fine sand	2.28
		1.87	95.99	2.13	Poorly sorted	Fine sand	2.03
		3.60	94.25	2.14	Poorly sorted	Medium sand	1.94
		1.89	93.09	5.01	Poorly sorted	Fine sand	2.27
		3.09	93.55	3.36	Poorly sorted	Fine sand	2.24
		2.34	92.65	4.99	Poorly sorted	Fine sand	2.31
		2.35	97.35	0.295	Moderately sorted	Medium sand	1.66
		1.34	94.87	3.788	Poorly sorted	Fine sand	2.10
	C	2.34	95.66	2.00	Poorly sorted	Fine sand	2.02
		1.34	93.70	4.95	Poorly sorted	Fine sand	2.65
		2.12	93.85	4.02	Poorly sorted	Fine sand	2.33
		2.50	91.70	5.84	Poorly sorted	Fine sand	2.49
		0.48	95.50	4.03	Poorly sorted	Fine sand	1.50
		1.93	90.73	7.34	Poorly sorted	Fine sand	2.85
		0.80	96.32	2.87	Moderately sorted	Fine sand	2.31
		0.20	95.23	4.57	Moderately sorted	Fine sand	2.88
		2.56	96.51	0.92	Moderately sorted	Medium sand	1.87
Early Dry	A	3.10	95.11	1.79	Poorly sorted	Fine sand	2.16
		6.42	88.02	5.56	Poorly sorted	Fine sand	2.19
		5.84	92.26	1.89	Poorly sorted	Medium sand	1.48
		1.56	95.5	2.94	Poorly sorted	Medium sand	1.77
		1.36	94.3	4.38	Poorly sorted	Fine sand	2.27
		5.95	92.6	1.43	Poorly sorted	Medium sand	1.08
		1.88	97.83	0.28	Moderately sorted	Medium sand	1.69
		1.46	94.84	3.69	Poorly sorted	Fine sand	2.22
		3.68	92.50	3.82	Poorly sorted	Medium sand	1.88
	B	1.84	94.43	3.72	Poorly sorted	Fine sand	2.09
		3.41	93.71	2.86	Poorly sorted	Medium sand	1.88
		2.33	96.28	1.39	Poorly sorted	Medium sand	1.70
		4.71	93.24	2.04	Poorly sorted	Medium sand	1.65
		0.84	94.20	4.96	Poorly sorted	Fine sand	2.21
		0.18	94.35	6.36	Poorly sorted	Fine sand	2.47
		0.02	99.90	0.01	Well sorted	Coarse sand	0.50
		4.91	94.13	0.96	Poorly sorted	Medium sand	1.52
		3.68	95.09	1.23	Poorly sorted	Medium sand	1.67
	C	5.40	93.14	1.45	Poorly sorted	Medium sand	1.27
		1.72	98.13	0.14	Moderately sorted	Medium sand	1.61
		1.60	96.09	2.30	Poorly sorted	Fine sand	2.08
		1.26	97.72	1.01	Poorly sorted	Medium sand	1.64
		1.72	95.26	3.02	Poorly sorted	Fine sand	2.08
		2.69	95.74	1.56	Poorly sorted	Medium sand	1.88
		6.36	92.14	1.50	Poorly sorted	Medium sand	1.56
		1.12	95.1	3.80	Poorly sorted	Medium sand	2.47
		3.25	95.37	1.38	Poorly sorted	Fine sand	2.05
Late Dry	A	2.46	93.91	3.62	Poorly sorted	Fine sand	2.07
		1.32	96.82	1.85	Poorly sorted	Medium sand	1.61
		3.24	93.1	3.66	Poorly sorted	Fine sand	2.19
		4.77	92.02	3.20	Poorly sorted	Medium sand	1.76
		4.39	94.17	1.43	Poorly sorted	Medium sand	1.45
		5.22	94.14	0.63	Poorly sorted	Medium sand	1.21
		7.24	91.55	1.20	Poorly sorted	Coarse sand	0.98
		2.77	95.24	1.98	Poorly sorted	Medium sand	1.28
		2.04	96.59	1.37	Poorly sorted	Medium sand	1.45
	B	23.2	75.7	1.09	Poorly sorted	Coarse sand	0.73
		8.08	89.14	2.77	Poorly sorted	Medium sand	1.56
		3.74	92.37	3.88	Poorly sorted	Medium sand	1.92
		10.71	88.16	1.13	Poorly sorted	Medium sand	1.31
		4.46	93.77	1.76	Poorly sorted	Medium sand	1.67
		5.13	92.48	2.39	Poorly sorted	Medium sand	1.60
		3.70	93.48	2.81	Poorly sorted	Medium sand	1.51
		4.70	93.39	1.90	Poorly sorted	Medium sand	1.36
	C	9.07	89.04	1.88	Poorly sorted	Medium sand	1.69
		2.85	93.49	3.65	Poorly sorted	Fine sand	2.14
		1.97	95.58	2.44	Poorly sorted	Fine sand	2.35
		1.45	95.85	2.70	Poorly sorted	Fine sand	2.36
		3.52	94.19	2.29	Poorly sorted	Medium sand	1.92
		2.98	95.03	1.98	Poorly sorted	Fine sand	2.11
		4.63	94.37	0.99	Poorly sorted	Medium sand	1.48
		5.06	93.18	1.75	Poorly sorted	Medium sand	1.79
		2.82	95.44	1.73	Poorly sorted	Fine sand	2.12
Early Rainy	A	0	17	83	Very poorly sorted	Fine silt	7.32
		0	15.85	84.15	Very poorly sorted	Clay	9.32
		0	10.06	89.94	Very poorly sorted	Clay	10.58
		0	13.73	86.27	Moderately sorted	Clay	12.01
		0	6.84	93.16	Very poorly sorted	Clay	10.07
		0	17.7	82.31	Unsorted	Coarse clay	8.45
		0	6.74	93.26	Very poorly sorted	Clay	11.27
		0	9.27	90.73	Well sorted	Clay	12.48
		0	7.03	15.76	Very poorly sorted	Clay	10.53
	B	0	13.12	86.88	Very poorly sorted	Clay	9.26
		0	7.46	92.54	Unsorted	Coarse clay	8.62
		0	6.76	93.24	Very poorly sorted	Clay	10.58
		0	8.59	91.41	Very poorly sorted	Clay	9.66
		0	10.27	89.73	Very poorly sorted	Clay	9.73
		0	2.88	97.11	Very poorly sorted	Clay	11.01
		0	7.67	92.63	Poorly sorted	Clay	11.73
		0	11.81	88.19	Moderately sorted	Clay	11.76
	C	0	13.13	86.87	Very poorly sorted	Clay	9.83
		0	12.74	87.26	Very poorly sorted	Clay	10.25
		0	13.28	86.72	Very poorly sorted	Clay	11.21
		0	20.67	92.34	Very poorly sorted	Clay	10.96
		0	7.66	88.63	Very poorly sorted	Clay	10.81
		0	11.36	84.97	Very poorly sorted	Clay	10.28
		0	15.03	93.69	Poorly sorted	Clay	11.78
		0	6.30	92.40	Very poorly sorted	Clay	12.14

### Biotic Data

**Figure 3:**
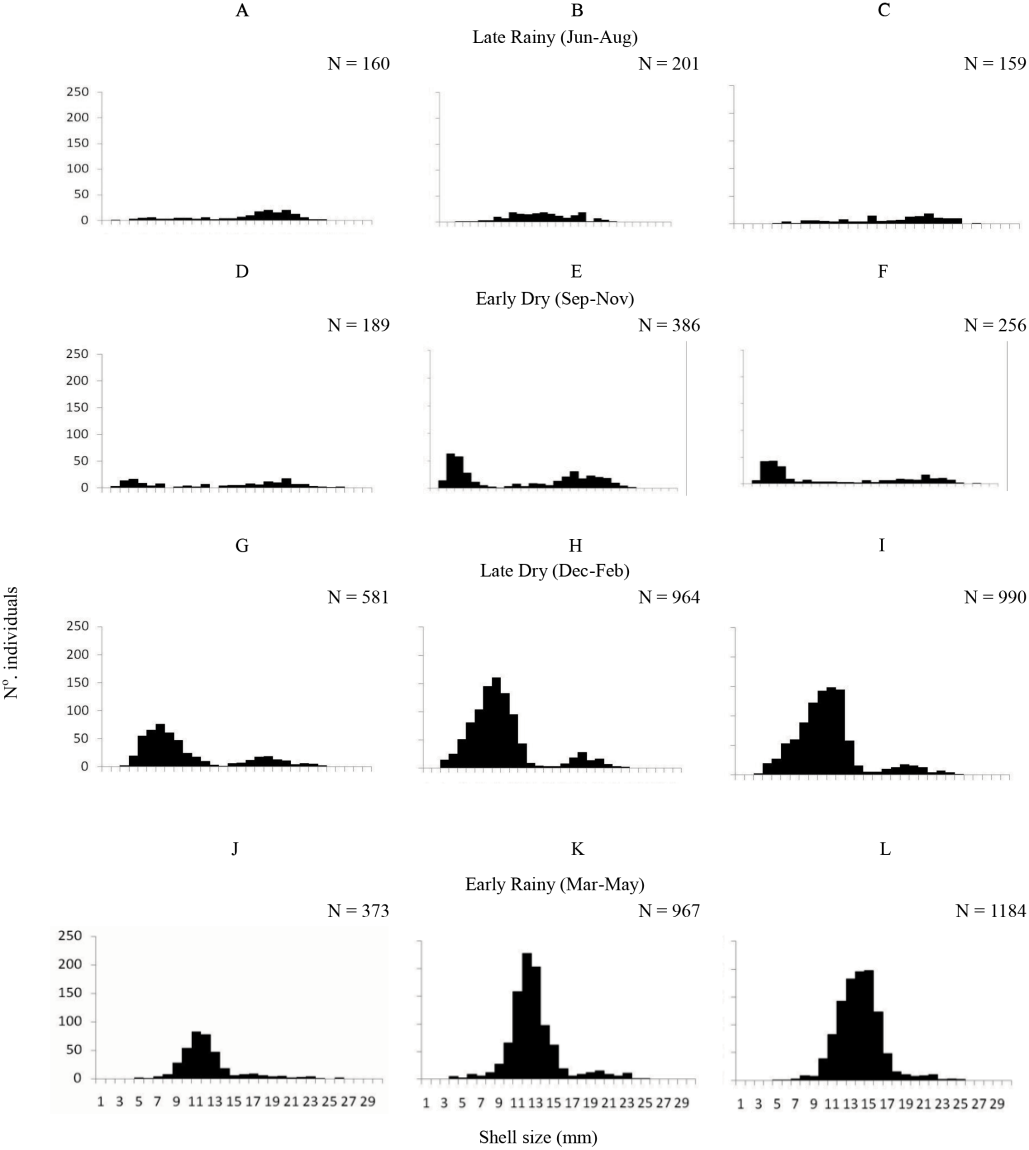
Shell length. Frequency distribution (number of individuals) of shell length (mm) of *Anomalocardia flexuosa* at three different habitats (habitat A (A, D, G and J); habitat B (B, E, H and L); habitat C (C, F, I and M)) on a tidal plain of the Goiana estuary during different seasons ((A–C) Late Rainy; (D–F) Early Dry; (G–I) Late Dry; (J–L) Early Rainy).

A total of 6,770 specimens of *A. flexuosa* were analysed and had shell between 2.20 and 28.48 mm (15.08 ± 4.08 mm). Recruitment was probably continuous, however higher numbers of recruits were registered in the late dry season (January –March) and in early rainy season as pointed below (Fig. 3). In respect to size, 1,303 organisms were collected from A, 1,163 were <20 mm of shell length. Adults (shell length > 20 mm) represented 9% (2,518) of the individuals in B; and 11.5% in C. Large number of recruits (shell length < 20 mm) were present in the late dry and early rainy seasons. Two-way ANOVA found significant differences (*p* < 0.05) were observed in the interaction between habitat and season *vs.* size.

**Figure 4:**
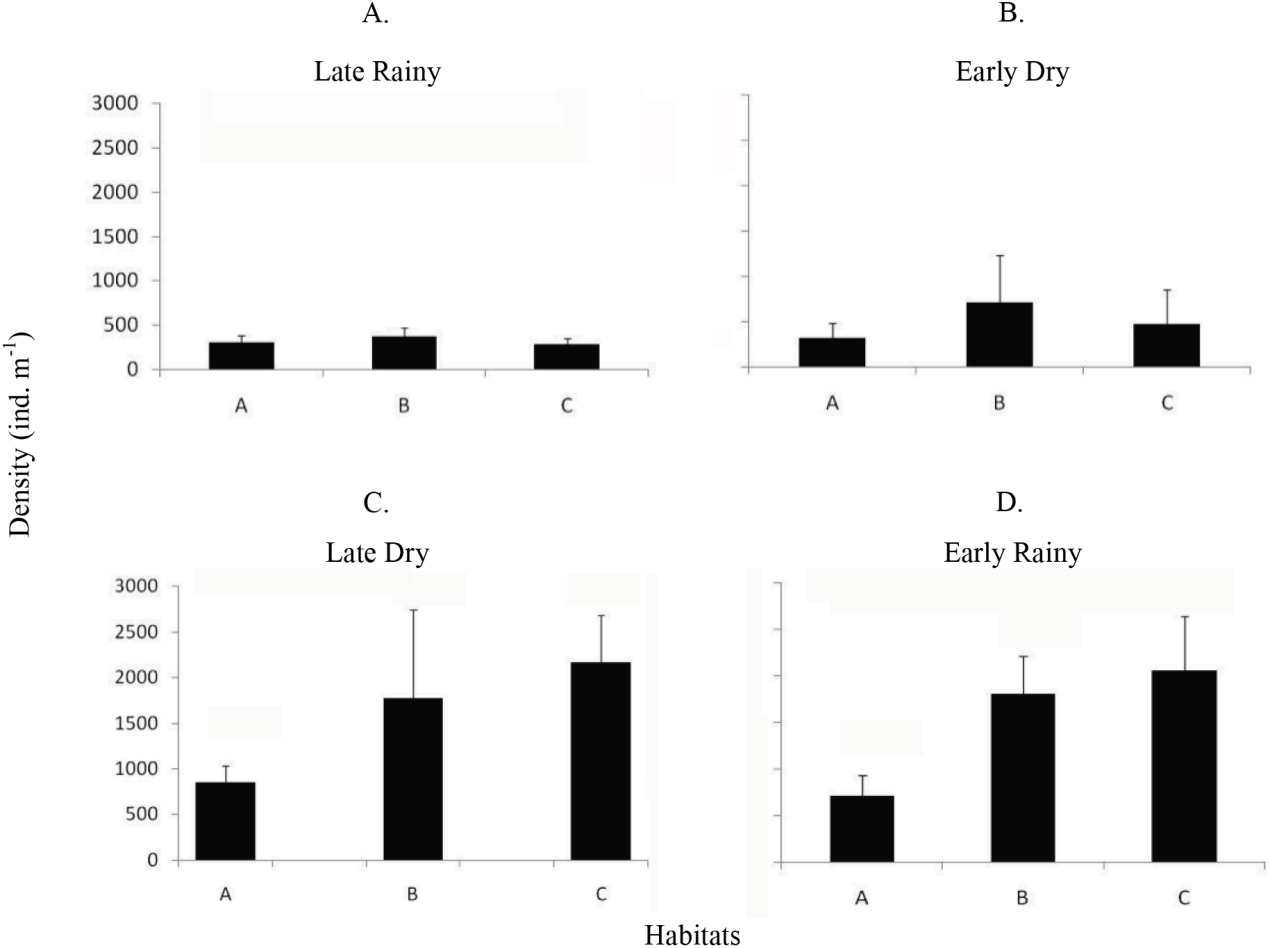
Density of *A. flexuosa*. Average and standard deviations density of *Anomalocardia flexuosa* (ind *m*^−1^) at a three different habitats (habitats A, B, C) of the tidal plain of the Goiana estuary along different seasons. (A) Late Rainy; (B) Early Dry; (C) Late Dry; (D) Early Rainy.

Mean density (ind m^−2^) was 1,600 ± 1,555 in late dry, 1,525 ± 1,389 in early rainy, 319 ± 259 in late rainy, and 496 ± 607 in early dry season. The maximum density value was lower in the late rainy season (0–1,170 ind m^−2^) and higher in late dry season (0–5,798 ind m^−2^) (Fig. 4).

**Figure 5:**
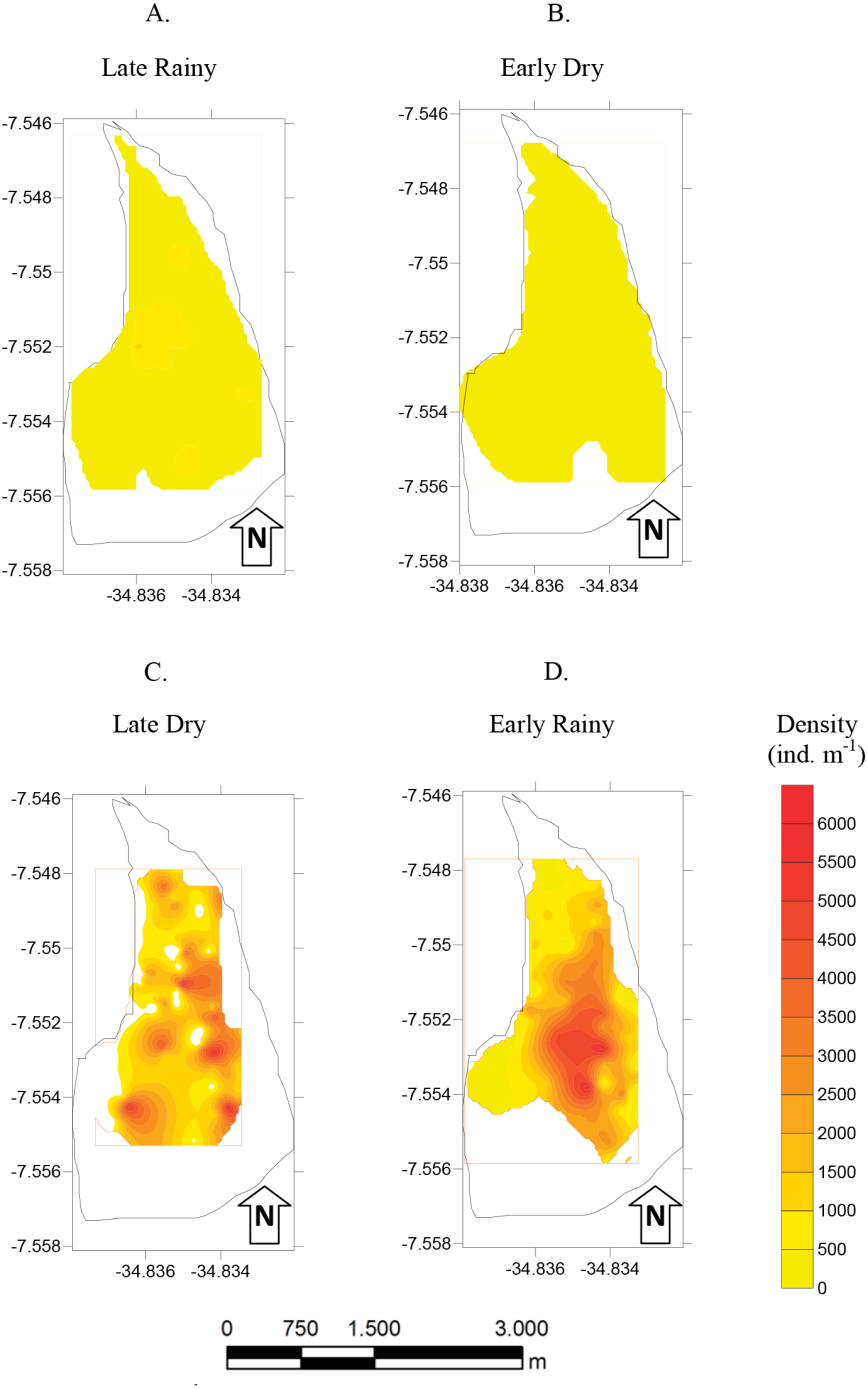
Map of density. Density (ind *m*^−1^) of *Anomalocardia flexuosa* at a tidal plain of the Goiana estuary along different seasons (A) Late Rainy; (B) Early Dry; (C) Late Dry; (D) Early Rainy.

The density habitats was lower in C (284 ± 70 ind m^−2^), A (305 ± 77 ind m^−2^) and B (369 ± 104 ind m^−2^) in the late rainy season (Fig. 5). In the early dry season, higher density values were found in habitats B (706 ± 525 ind m^−2^), C (471 ± 376 ind m^−2^) and A (314 ± 169 ind m^−2^). Density increased in the late dry season when there were a high number of recruits settling on the tidal plain (Fig. 5). Recruitment increased density in habitats C (2,170 ± 971 ind m^−2^) and B (1,777 ± 961 ind m^−2^) (Fig. 5). In the early rainy season, C (2,060 ind m^−2^) remained as the most densely populated habitat followed by habitats B (1,800 ind m^−2^) and A (706 ind m^−2^).

**Figure 6:**
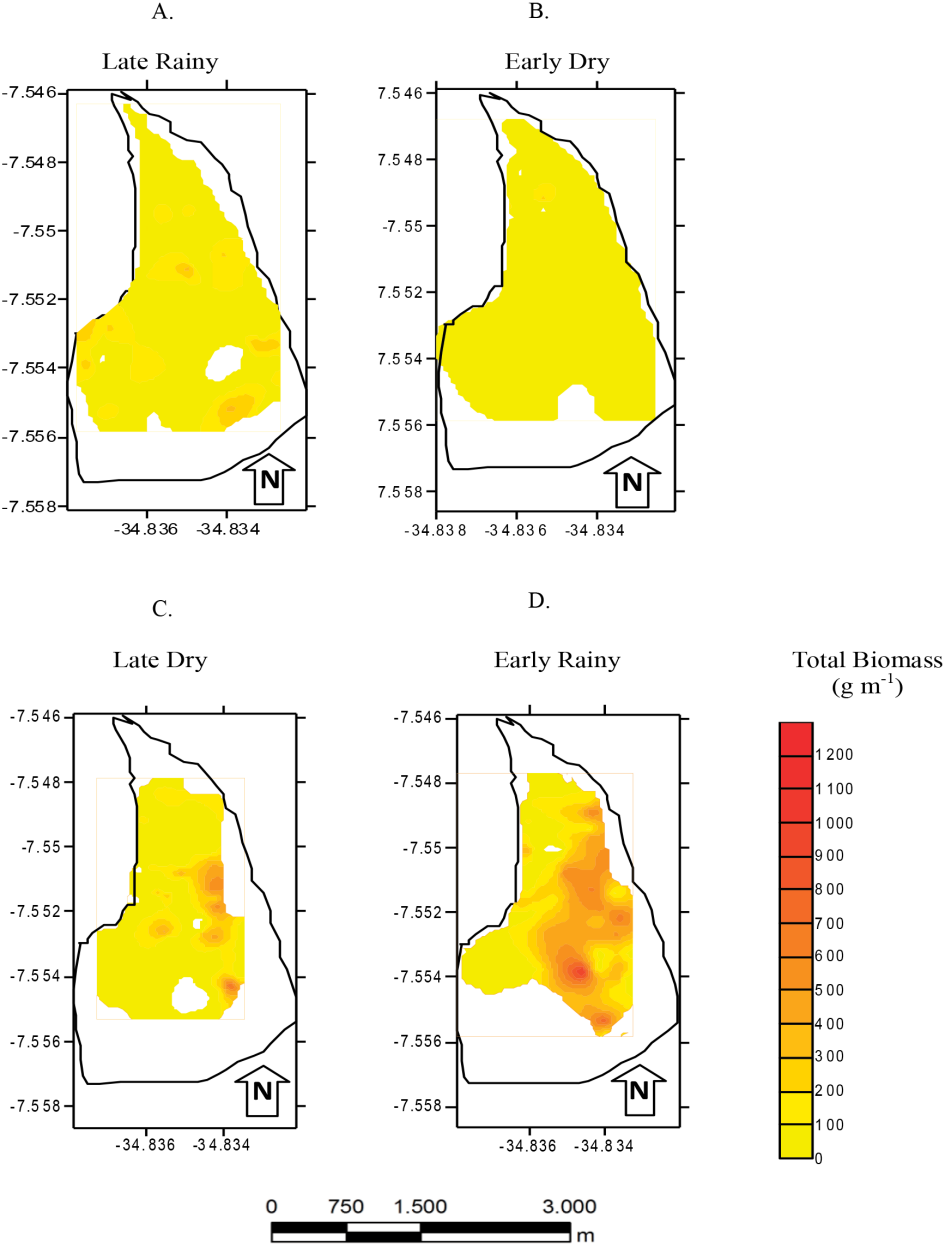
Map of biomass. Total biomass (g *m*^−2^) of *Anomalocardia flexuosa* at a tidal plain in the Goiana estuary along different seasons. (A) Late Rainy; (B) Early Dry; (C) Late Dry; (D) Early Rainy.

Total biomass was higher in early rainy season (221.0 ± 231.44 g m^−2^) (Fig. 6) in all habitats of the tidal plain (A, B and C). Seasonal Biomass means were 75.6 ± 90.9; 57.34 ± 97; 221 ± 231.4 and 23.46 ± 34.39 g m^−2^ for late rainy, late dry, early rainy and early dry season. The highest Biomass was observed in habitats B (95.5 g m^−2^) and C (133.25 g m^−2^) (Fig. 6).

**Figure 7:**
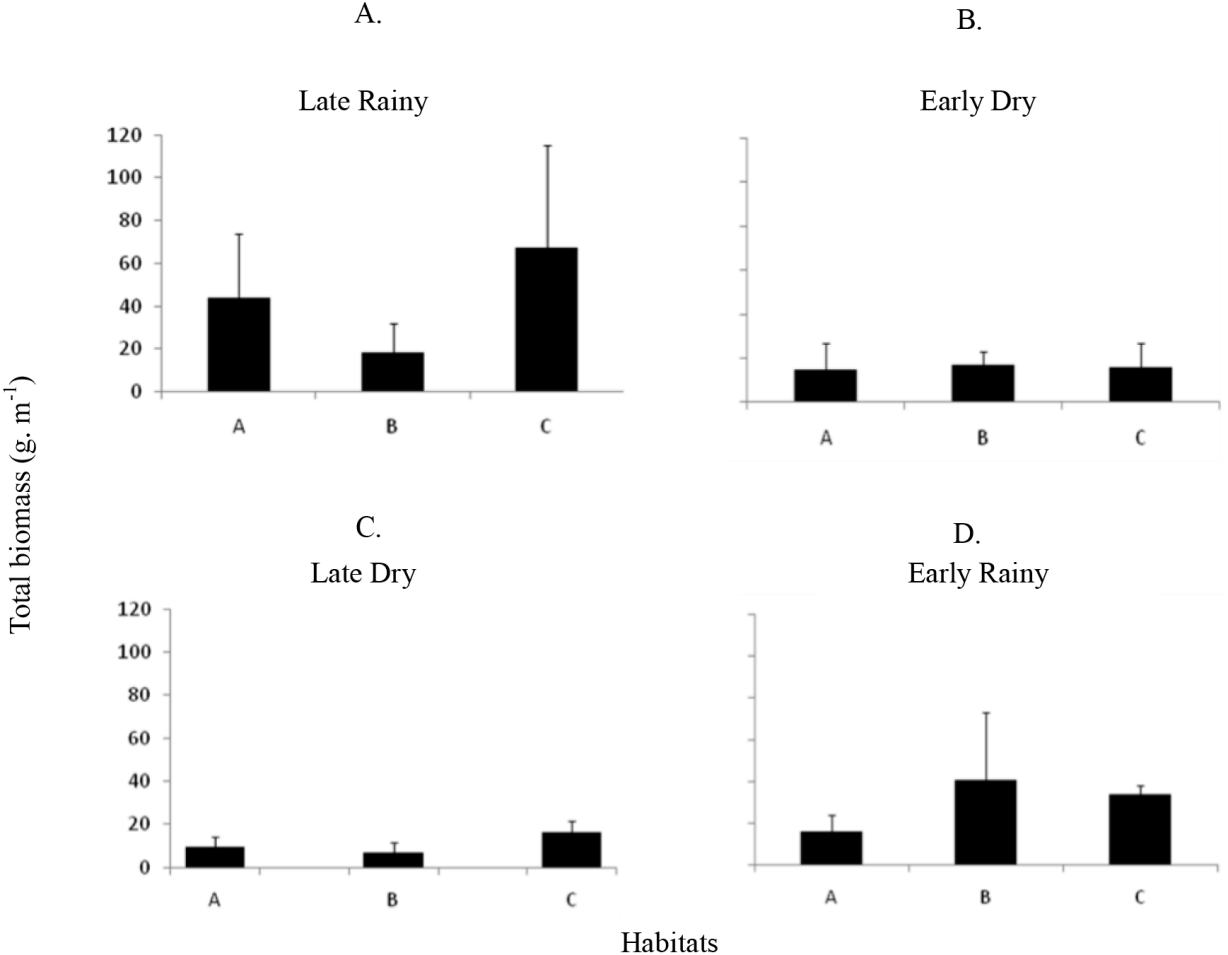
Average and deviations biomass. Average and deviations biomass (shell length > 20 mm) (g *m*^−2^) of *Anomalocardia flexuosa* at three habitats of a tidal of the Goiana estuary along different seasons. (A) Late Rainy; (B) Early Dry; (C) Late Dry; (D) Early Rainy.

Seven hundred and ninety individuals of *A. flexuosa* had shell lenght >20 mm. However, adult biomass was lower (0 to 308.5 g m^−2^) than total biomass (Fig. 7). Adult Biomass (shell length > 20 mm) did not present significant differences among habitats (*p* > 0.05). Seasonal adult Biomass (shell length > 20 mm) peaked in the late rainy season (43.4 g m^−2^) when it was responsible for most of TB (90.8, 29.3 and 75.4%) in A, B and C, respectively (Fig. 7). Early rainy season adult Biomass represented between 56 to 78% for estimated TB. Late dry season was when the lowest mean adult Biomass (shell length > 20 mm) occurred (10.8 g m^−2^ ± 4.32). The adult biomass (shell length > 20 mm) showed differences in relation to season (*p* < 0.01), when early and late dry seasons. The interaction between season and habitat showed significant differences (*p* < 0.05): habitat B during early dry was different from all other habitats and seasons.

**Figure 8:**
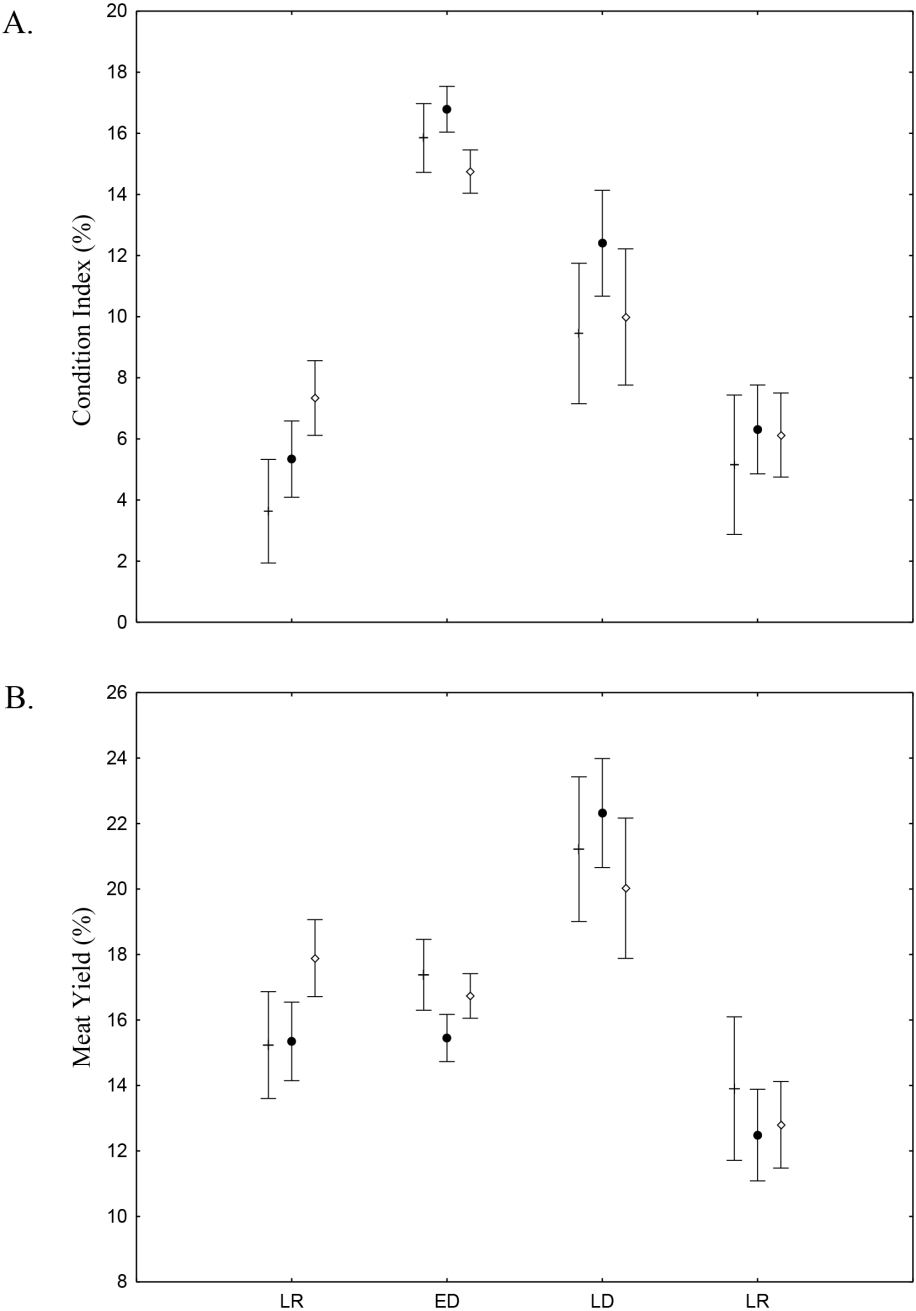
Meat Yield and Condition Index. Meat Yield (A) and Condition Index (B) average and standard deviations of *Anomalocardia flexuosa* > 10 mm at the tidal plain (□ = *A*; • = *B*; □ = *C*) at Goiana estuary along different seasons (LR: Late Rainy; ED: Early Dry; LD: Late Dry and ER; Early Rainy). *N* = 4736 ind. (A: 883; B: 1880; C: 1733), *p* ≤ 0.05. Interaction season vs. habitat for MY (*F*_(6,4,724)_ = 2.7116, *p* = 0.01248) and CI (*F*_(6,4,724)_ = 3.9232, *p* = 0.00065).

There was found spatio-temporal variation in Condition index (CI) and Meat Yield (MY) of *A. flexuosa* along the year. CI showed significant differences (*p* < 0.05) for season and (Fig. 8A; Table 4). MY were 16.9  ± 10.5% in habitat A, 15.7 ± 10.8% in habitat B and 16.6 ± 13.6% in C (Fig. 8B). Meat yield was significantly correlated to the habitat *vs.* season interaction (*p* < 0.05).

## Discussion

Rainfall patterns determine the occurrence of well-defined seasons (Barletta & Costa, 2009). Salinity decreases in the early rainy at Goiana Estuary, ranging from 8 to 36 (Dantas et al., 2010). Water temperature showed the same seasonal trend, with lower temperatures occurring in the late rainy (26 °C) (Dantas et al., 2010). Rainfall is also responsible for the variation of interstitial water salinity along different seasons. An increase in the relative abundance of *A. flexuosa* was evidenced in the three habitats during the early rainy season. The grain size changes (sand to clay/silt) can explain the abundance of *Anomalocardia flexuosa* during this period. The fine grain predominance favored the *A. flexuosa* settling in the different habitats. These results corroborate Rodrigues et al. (2008) that showed an increase of abundance in this tidal plain where the fine grain size (clay/silt) was dominant.

In this tidal studied in the Goiana estuary there were two cohorts of 3–16 mm and 17–23 mm in shell length. This is probably the shell size fishers select either by hand or with rudimentary tools. The adult organism’s ocurrence was lower along seasons. The size distribution shows the presence of more than one cohort, as demonstrated in studies of species elsewhere. *Anomalocardia flexuosa* recruitment was probably a reproductive peak and recruitment phases during early dry and late dry seasons, respectively. Adult biomass values (shell length > 20 mm) were lower during late dry season and higher in late rain season (90% total biomass). Similar results were found for Ceará State (Araújo & Rocha-Barreira, 2004). The occurrence of a young cohort (shell length < 20 mm) indicates continuous reproduction and a short life cycle. Population dynamics works showed that *A. flexuosa* longevity ranges between 1.5 to 4.6 years (Monti, Frenkiel & Möueza, 1991; Souza, 2007; Rodrigues et al., 2008). Therefore, five years can be considered as the recovered time for overfished areas, after pressure by capture of this species is diminished or has ceased. Current economic and ecological models proposed that the reduction of fishing effort (e.g., elimination of overfishing and reduced habitat disturbance) could positively affect the ecosystem and allow economic and social welfare gains (Wang et al., 2016). This would be the case here, since a collapse of the mollusc population might entail grave social risk of income reduction for more than 500 families.

The settling phase was concentrated during austral summer (September to January), when fishers exert the greater pressure on *A. flexuosa* stocks, reducing the mature adult population (Silva-Cavalcanti & Costa, 2009). This could help settling by increasing available space for recruits, but also sediments turbation by hands and tools can bury recruits and kill them by suffocation, since they would not gain easy access to sediment surface during high tide to filter and feed. According to Boehs, Absher & Cruz-Kaled (2008), more available space for recruits can only favour settlers in the late dry season, when reduction of rainfall and reduced predation allow time and space for them to grow.

Abundance in an exploited bivalve population depends on the balance between inputs (reproduction/recruitment and growth) and outputs (mortality and fishery removals) of adult/mature individuals. Reproduction and subsequent succesful recruitment are crucial for population sustainability (Magalhães, Freitas & Montaudouin, 2016). However, water temperature is only one of the myriad components determining recruitment success (Magalhães, Freitas & Montaudouin, 2016). In the case of the Goiana estuary, early maturation due to more constant water temperature, even through the rainy season, can also be playing an important role.

**Table 4:** Kruskal–Wallis analysis for density (ind m^−2^), Total Biomass, total and adult Biomass (>20 mm) (g m^−2^). ANOVA multifactorial was used for other variables. Differences between habitats *vs.* season were determined by a post hoc Bonferroni test. NS, Non significant; LR: Late Rainy; ED: Early Dry; LD: Late Dry and ER; Early Rainy; A; B and C are habitats of a tidal plain at Goiana estuary.

	Variation Source		
Variable	Season (1)	Habitat (2)	Interations
Shell lenght (mm)	^**^LR**ED** LD**ER**	^**^A B C	1 × 2^**^
Total biomass (g m^−2^)	^**^LR**ED** LD*IC ER*	NS	NS
Adult biomass (shell length >20 mm) (g m^−2^)	^**^LR**ED LD***ER*	NS	1 × 2^**^
Density (ind m^−2^)	^*^LR ED***LD*ER**	NS	1 × 2^**^
Condition index (CI)	^**^LR ED**LD***ER*	^*^A*B****C***	1 × 2^**^
Meat yield (MY)	^*^LR EDLD*ER*	NS	1 × 2^*^

**Notes.**

* *P* < 0.05.

** *P* < 0.01.

Adults (shell length > 20 mm) are preferred by fishers during summer due to meat yield and processing speed (Silva-Cavalcanti & Costa, 2009). In addition, mussel fishing and consumption increases during austral summer, adding non-traditional workers to the fishing effort, which results in unsustaintable scenarios (Silva-Cavalcanti & Costa, 2009). Population mean size (shell length ∼15 mm) at Goiana estuary and other sites suggests excessive harvesting and catch of individuals bellow maturation size (Araújo, 2001; Arruda-Soares, Schaeffer-Novelli & Mandelli, 1982; Boehs et al., 2003). Long-term assessments (∼10 to 20 years) suggest that unsustainable practices in the fisheries of *A. flexuosa* affect life quality of traditional coastal populations (Silva-Cavalcanti & Costa, 2009). Management polices can help reduce overfishing, detain decreases of the population average shell size and finally help increase the abundance of reproductive individuals and recruitment.

The performance of MPAs aimed at traditional fisheries activities depends on several biological and ecological factors related to the target species, such as substrate availability, quantity and quality of food resources (i.e., microalgal films) and the degree of overlap with other species from the same ecological and trophic guilds (Riera et al., 2016). A factor of utmost importance is regulations enforcement to prevent unplanned harvesting, too common in intertidal areas because of the readly available target species and the lack of refuge (Riera et al., 2016). Tidal plain living resources may reflect an erroneous impression of commons, as described in community succession models (Gerwing et al., 2016). Optimum size (shell length) to haverst *A. flexuosa* is suggested at 20 mm (Arruda-Soares, Schaeffer-Novelli & Mandelli, 1982; Barreira & Araújo, 2005) when gonadal development is reached. However, there are no *A. flexuosa* above 20 mm (shell length) in Acupe (Bahia State) (Martins & Souto, 2006), for example. Survival of human populations overcomes ecological concerns (Martins & Souto, 2006) at Goiana estuary, where most families are below the poverty line, as reflected in very low Human Development Index (IDH) and Education Development Index (IDE) (CPRH, 2003). Thus, *A. flexuosa*, finfish, mangrove firewood, mangrove crabs and lobsters are exploited without any short or long-term concerns (Guebert-Bartholo et al., 2011). Shell size of *Anomalocardia flexuosa* in the Goiana estuary (mean 15.08 mm ± 4.08 of shell length) was lower than in other areas with fishing activity (Monti, Frenkiel & Möueza, 1991; Mouëza, Gros & Frenkiel, 1999; Schio, Souza & Pezzuto, 2007; Figueredo & Lavrado, 2007; Mattos, Cardoso & Caetano, 2008; Lima, Barbosa & Correia, 2007; Rodrigues et al., 2013; Morsan, 2007; Boehs et al., 2003). Thus, it is possible that the population will soon collapse and no longer be able to sustain its fisheries production chain.

TB at Goiana estuary changed along seasons. However, the population structure can be relatively coupled from the structuring influences of biotic and abiotic factors in this system. High concentration of resources sustain high density of infauna, and limit exploitative competition reflecting particulary events as namely a “first come, first served” process (Gerwing et al., 2016; Magalhães, Freitas & Montaudouin, 2016). The design of the MPA should consider the connectivity among biomass exchanges sites and surrounding areas (Riera et al., 2016).

Density data was compatible with areas considered in expansion or overfishing phases (Castilla & Defeo, 2001; Souza, 2007; Schio, Souza & Pezzuto, 2007). Pirajubaé Marine Protected Area—MPA density values of *A. flexuosa* ranged from 97 to 203 ind m^−2^ for shell length of 19–27 mm and biomass 795 g m^−2^ (Schio, Souza & Pezzuto, 2007). In the present work, biomass ranged from 19 to 338 g m^−2^, well bellow Pirajubaé and thus, according to these authors, problaby in the overfishing phase.

Shell size, total wheight and CI were different along the three sampling habitats. Abiotic data were not spatially different. However, capture was different among sampling habitats, A and B being the most used. It is possible that sexually mature individuals from lesser non-exploited areas (ex. C and sub-tidal reaches), kept stocks throughout the Goiana estuary.

The primary effects of size-selective harvesting are the overall reduction in body size and an increased mortality rate of harvested species, but other concomitant factors are associated, such as reproductive investment and changes in growth rate and relative fecundity (Riera et al., 2016). Thus, population changes from human intervation are difficult to assess because there is no information about any unexploited site or control site to use as reference. To prevent these processes, it is necessary to develop effective management strategies to recover limpet populations, such as regulations and the creation of no-take zones.

Condition Index (CI) and Meat Yield (MY) values indicate conversion of glycogen in gametes, sexual maturation and spawning, as well as reflecting the stress and nutritional state of *A. flexuosa* (Absher & Christo, 1993; Aswani et al., 2004; Christo, 2006). Higher temperature and interstitial water salinity, during early and late dry season, stimulated spawning (Christo, 2006). In this time, in Goiana estuary, gonadal development was over or partial, thus magnifying the weight of the individual and these two indexes. However, MY was higher than CI during late rainy season when lower salinity values stressed animals. MY and CI were lowest during early rainy season probably due to spawning. These values were significatly different (*p* ≤ 0.05) and suggest that interstitial water salinity and temperature influencied MY and CI. Fisher perceived MY changes along the year and try to profit from occasions when it is favourable due to gonadal maturation, thus upsetting the population right at its reproductive peak (Silva-Cavalcanti & Costa, 2009).

## Conclusion

The Goiana estuary has an *Anomalocardia flexuosa* stock composed mainly of juveniles (<20 mm shell lenght) that its highest biomass during the main recruitment season. Spawning occurs in the late rainy season and might extend up to the late dry season. Interstitial water sanility, temperature and grain size influenced *A. flexuosa* abundance, and are thus important variables for determining abundance and distribution of this species in the region. Therefore, regulating fisheries might help this overfished population in different ways, not only by reducing adult/mature biomass removal, but also leaving sediments undisturbed at no-take zones and for longer periods.

Seasonal differences were observed for biomass and density, with the early rainy season presenting higher values. Theses values suggest that *A. flexuosa* is overfished. The reprodution peak was in the late rainy season (June to August). Reproductive cycle and density data are important variables for the evaluation of the fishing stocks as well as for the design and assessment of resource management plans and instruments. Interannual and interdecadal studies about variations in population parameters at control sites will be important to create standards to compare with sites under anthropogenic interference. These investigations can better estimate, in a medium term (3–5 years), the potential recovered of well-managed stocks. This information can then help in deciding about location and habilitation of new MPAs.

##  Supplemental Information

10.7717/peerj.4332/supp-1 Supplemental Information 1 Raw data Field files.

## Additional Information and Declarations

### Field Study Permissions

The following information was supplied relating to field study approvals (i.e., approving body and any reference numbers): Field experiments were approved by the Federal University of Pernambuco and Enviroment Ministry of Brazil (MMA-IBAMA).

### Data Availability

The following information was supplied regarding data availability: The raw data has been supplied as a Supplementary File.
